# MoS_2_ and CdMoS_4_ nanostructure-based UV light photodetectors†

**DOI:** 10.1039/d1na00326g

**Published:** 2021-07-21

**Authors:** Mahendra S. Pawar, Sunil R. Kadam, Bharat B. Kale, Dattatray J. Late

**Affiliations:** Physical and Material Chemistry Division, CSIR – National Chemical Laboratory Pune 411008 Maharashtra India datta099@gmail.com djlate@mum.amity.edu; Academy of Scientific and Innovative Research (AcSIR) Ghaziabad 201002 India; Centre for Materials for Electronics Technology (C-MET), Department of Electronics and Information and Technology (DeitY) Pune 411008 Maharashtra India bbkale@cmet.gov.in; Centre for Nanoscience & Nanotechnology, Amity University Maharashtra Mumbai-Pune Expressway, Bhatan, Post – Somathne, Panvel Mumbai Maharashtra 410206 India

## Abstract

We have developed MoS_2_ nanosheets and CdMoS_4_ hierarchical nanostructures based on a UV light photodetector. The surface morphologies of the as-prepared samples were investigated *via* field emission scanning electron microscopy (FESEM) and transmission electron microscopy (TEM). The performance parameters for the present photodetectors are investigated under the illumination of UV light having a wavelength of ∼385 nm. Upon the illumination of UV light, the CdMoS_4_-based photodetector device showed a better response to UV light compared to the MoS_2_ device in terms of photoresponsivity, response time (∼72 s) and recovery time (∼94 s). Our results reveal that CdMoS_4_ hierarchical nanostructures are practical for enhancing the device performance.

## Abstract 2

We have developed MoS_2_ nanosheets and CdMoS_4_ hierarchical nanostructures based on a UV light photodetector.

## Graphical abstract

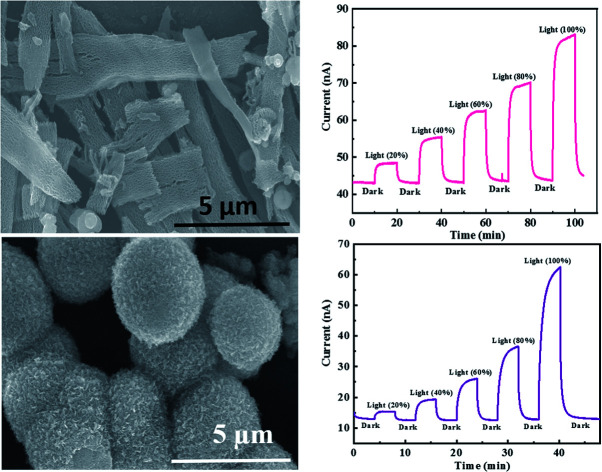

## Introduction

Photodetectors based on nanostructured materials are the significant components in nanoelectronic and optoelectronic devices.^1–4^ Photodetectors with quick photoresponse and high photoresponsivity are in much demand for real-world applications such as optical imaging and communication.^2,5^ To date, photodetectors from numerous nanomaterials (0D, 1D, and 2D) with remarkable response time and high responsivity have been reported to have excitation wavelengths ranging from ultraviolet to near-infrared region.^6–12^ For the development of high-performance photodetectors, semiconducting materials with an appropriate bandgap and nanostructured morphology are highly desirable and important. Although two-dimensional (2D) graphene has attracted significant interest in numerous nanoelectronic systems due to its outstanding electronic, thermal, and mechanical properties, further development has been restricted in optoelectronic devices due to its zero bandgap or semimetallic nature.^13^ In previous reports, researchers have developed a strategy to create a bandgap in graphene and utilized it for broadband photodetector application.^14^ However, the presence of favourable bandgap in other 2D inorganic layered materials such as MoS_2_, WS_2_, MoSe_2_, SnSe_2_ and black phosphorous suggests great promise in the fabrication of large scale photodetector devices.^15–23^ These 2D materials also possess a layer-dependent tunable bandgap, *i.e.*, direct bandgap in monolayer and indirect bandgap in the bulk form. It makes these materials a potential candidate in numerous applications such as field effect transistors, solar cells, gas sensors, and energy storage devices.^24–28^ Due to the strong light absorption in the visible to near-infrared region, MoS_2_ is widely used in optoelectronic systems. In 2012, Zhang *et al.* reported a single-layer MoS_2_ phototransistor for the first time and obtained maximum photoresponsivity of ∼7.5 mA W^−1^ at an applied gate voltage (*V*_g_, 50 V).^12^ Jason *et al.* reported near-infrared photodetection using bilayer MoS_2_, where they have injected hot electrons into MoS_2_ in order to originate sub-bandgap photocurrent results into photogain of the order of ∼10^5^. This photogain leads to a photoresponsivity of 5.2 A W^−1^ at 1070 nm, which is much higher than that of Si-based photodetectors.^29^ Also, another report from Zhai *et al.* on monolayer MoS_2_ coupled with an organic molecule showed a fast response time of 8 ms and maximum photoresponsivity of ∼430 A W^−1^ after Al_2_O_3_ passivation.^30^ In addition, reports on MoS_2_-based photodetectors show that it reduces the recombination rate of charge carriers along with photoabsorber, thus leading to an increase in the photocurrent.^31–33^ Herein, we report the fabrication of 2D MoS_2_ nanosheets and 3D CdMoS_4_ nanoflowers based on UV light photodetectors. The solvothermal route was implied for the synthesis of these structures, followed by structural and morphological investigation. We also demonstrated the device performance of these samples under UV light illumination and their cyclic response.

## Experimental method

### Synthesis of MoS_2_ nanosheets

Initially, we dissolved 2 mmol of ammonium molybdate in (40–50 ml) methanol with the help of a stirrer. To this mixture, we added dissolved thiourea in methanol dropwise, followed by stirring for 15 min. The whole solution was then transferred into a Teflon-lined stainless steel autoclave at 150 °C for 48 h. The precipitate was obtained using a Whatman filter paper, followed by washing with ethanol several times and then heated in an oven at 80 °C for 4 h. The MoS_2_ powder sample was then annealed at 400 °C for 4 h in an N_2_ atmosphere.

### Synthesis of CdMoS_4_ nanoflowers

For the CdMoS_4_ nanoflowers synthesis, we have taken 2 mmol of cadmium nitrate and dissolved it in 40–50 ml methanol. Also, we prepared a 20 ml ammonium molybdate solution by dissolving a ammonium molybdate precursor in methanol with the help of a stirrer and continued for 10 min. To this solution, we added a dissolved cadmium nitrate solution dropwise under constant stirring for 10 min. Next, dissolved thiourea was added dropwise to it, and the solution mixture was stirred further for 15 min. The solution was then transferred into a Teflon-lined stainless steel autoclave at 150 °C for 48 h. After completion of the reaction, the reactor was allowed to cool down to room temperature naturally, followed by washing the product with distilled water and filtered using a Whatman filter paper. The precipitate obtained was washed with ethanol several times and then heated in an oven at 80 °C for 4 h. The CdMoS_4_ powder sample was then annealed at 400 °C for 4 h in an N_2_ atmosphere.

Both the products were further analysed using numerous microscopy and spectroscopy techniques. The details on the synthesis of MoS_2_ nanosheets and CdMoS_4_ nanoflowers were reported previously.^34^

### Material characterizations

The structural investigations were carried out *via* X-ray powder diffraction technique (XRD-D8, Advance, Bruker-AXS) and Raman spectrometry (HR-800, Horiba Jobin Yvon, France) at an excitation laser wavelength of 632.8 nm with a power density of 6.37 × 10^7^ W cm^−1^.^2^ The surface morphologies of the as-prepared samples were characterized using a field emission scanning electron microscope (FESEM, Hitachi, S-4800) and transmission electron microscope (TEM, JEOL, 2010F Instrument).

### Device fabrication and electrical measurements

The devices were fabricated on a indium tin oxide (ITO)-coated glass substrate with a source-drain separation of 500 µm. The central region of the conducting surface was etched with the zinc dust and concentrated HCl treatment. Then, we drop-casted our prepared material in this nonconducting region of the ITO substrate. Further, we annealed the device at 200 °C for 8 h in vacuum in order to obtain good adhesion of the as-prepared material with the glass substrate. The electrical characterizations of the fabricated devices for MoS_2_ and CdMoS_4_ samples were carried out using a Keithley 2612A source meter, which was connected to a computer through a GPIB 288A interface. All the measurements were done at room temperature.

## Results and discussion

### XRD of MoS_2_ and CdMoS_4_

**Fig. 1:**
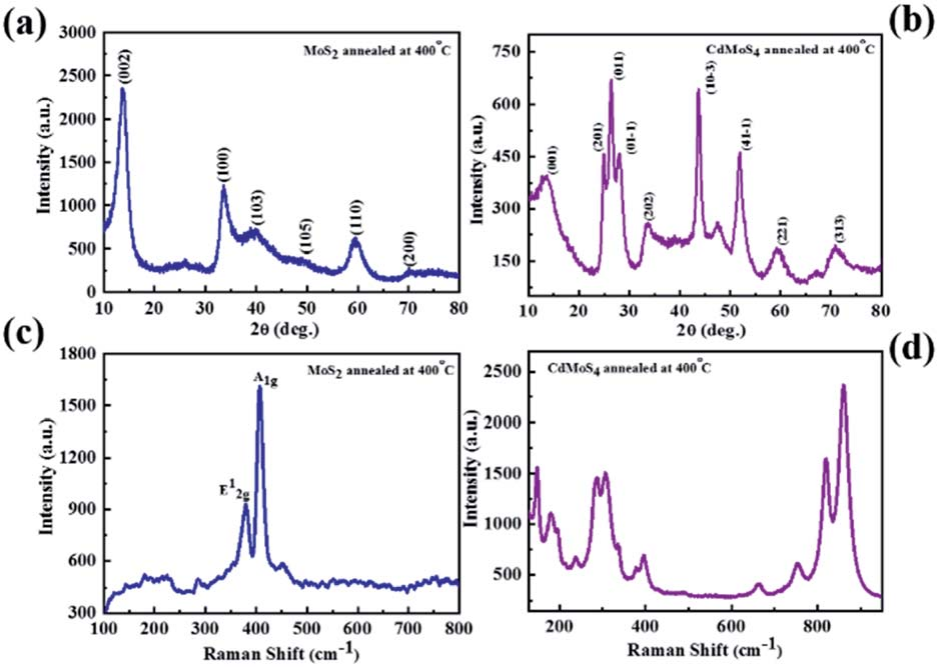
XRD pattern of (a) MoS_2_ and (b) CdMoS_4_ samples annealed at 400 °C, Raman spectra of (c) MoS_2_ and (d) CdMoS_4_ samples annealed at 400 °C.

The phase formation of the as-synthesized MoS_2_ and CdMoS_4_ samples were confirmed by XRD and depicted in Fig. 1(a and b). The XRD patterns of the MoS_2_ samples synthesized at 400 °C for 4 h are shown in Fig. 1(a), which match with the JCPDS data card no. 00-009-0312, confirming the formation of a pure hexagonal phase of layered MoS_2_ and match well with the earlier report.^35^Fig. 1(b) shows the XRD pattern for the CdMoS_4_ sample annealed at 400 °C for 4 h. Indexing of the annealed CdMoS_4_ sample was carried out using the Powder 4-DICVOLE software, which validated the formation of the monoclinic structure of CdMoS_4_.

### Raman spectroscopy of MoS_2_ and CdMoS_4_

In view of this, the as-prepared samples were characterized *via* Raman spectroscopy and are shown in Fig. 1(c) and (d). The two Raman peaks assigned to the MoS_2_ sample correspond to the E^1^_2g_ (in-plane) and A_1g_ (out-of- plane) vibration mode, as shown in Fig. 1(c).

These two Raman modes E^1^_2g_ and A_1g_ appear at 382.5 and 406 cm^−1^, respectively. The separation of 27.56 cm^−1^ between these two peaks specified the presence of the few-layer nature of MoS_2_. The intense Raman peaks at 125, 150, 182, 238, 285, 307, 398, 664, 754, 819, 860 and 890 cm^−1^ were observed for annealed CdMoS_4_ sample shown in Fig. 1(d).

### FESEM and TEM of MoS_2_

**Fig. 2:**
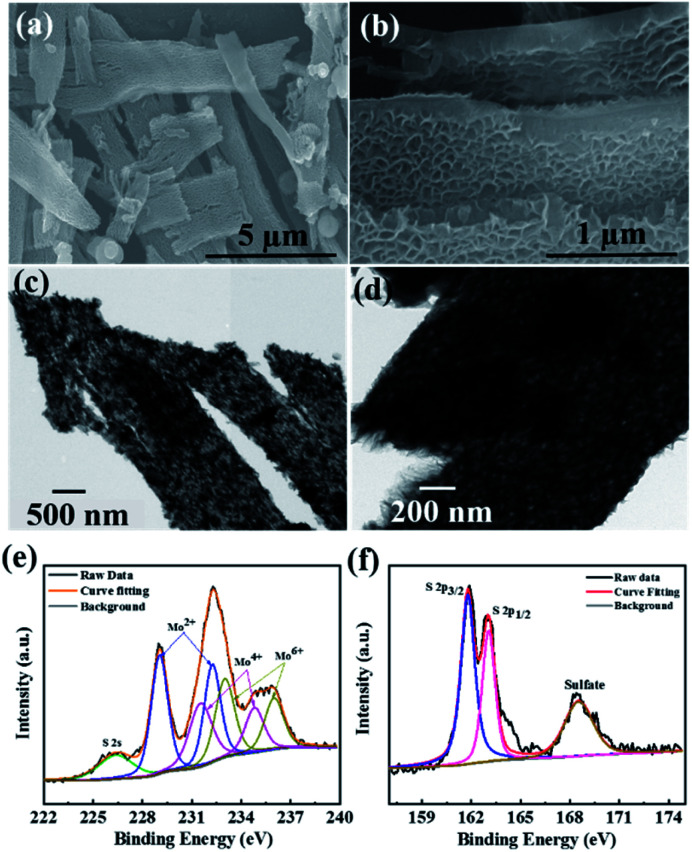
(a and b) FESEM images, (c and d) TEM images and (e and f) deconvoluted XPS spectra for Mo 3d and S 2p elements of the MoS_2_ samples annealed at 400 °C.

**Fig. 3:**
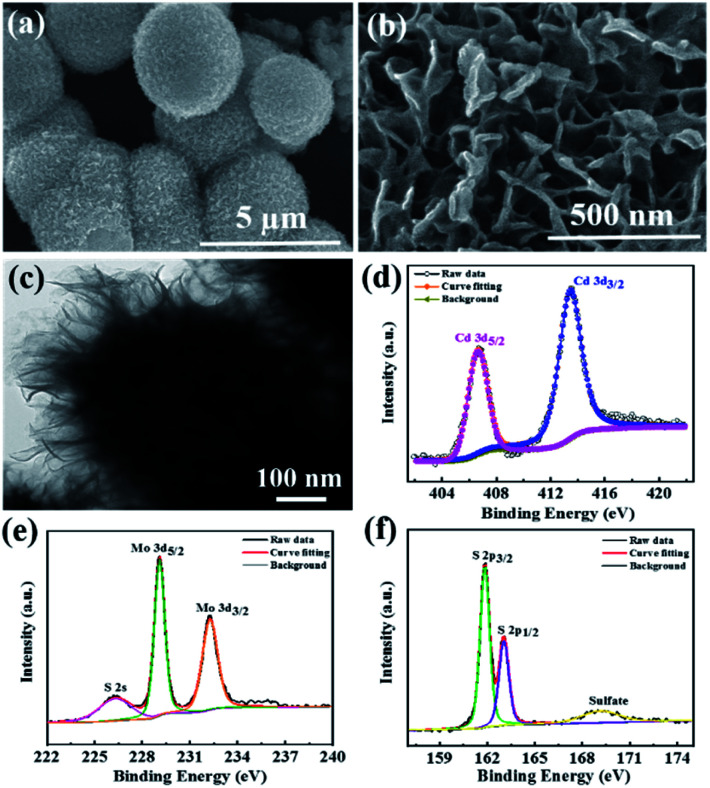
(a and b) FESEM images, (c) TEM image and (d–f) deconvoluted XPS spectra for Cd 3d, Mo 3d, and S 2p of the CdMoS_4_ nanoflowers annealed at 400 °C.

In order to investigate the surface morphology of the as-synthesized MoS_2_ and CdMoS_4_ samples, FESEM analysis was carried out and the results are depicted in Fig. 2 and 3, respectively. We observed some brake petals of MoS_2_ sheets of different sizes located on the long sheet shown in Fig. 2(a and b). The long honeycomb-like sheets measured up to 10 µm and the thickness was found to be ∼200 nm (Fig. 2(a and b)). The pore size of the honeycomb sheet is 40–50 nm, and the thickness of the single cell is ∼10 nm. The TEM image depicted in Fig. 2(c and d) represents the sheet-like structure of MoS_2_ and matches well with the FESEM results. The MoS_2_ samples were entirely honeycomb-like sheets without aggregation.

### XPS of MoS_2_

The X-ray photoelectron spectroscopy (XPS) was carried out for MoS_2_ sheets, and core-level XPS spectra for Mo 3d and S 2p are shown in Fig. 2(e and f). Fig. 2(e) displays few characteristic peaks corresponding to the orbital of Mo^2+^, Mo^4+^ and Mo^6+^ observed at 232.29/229.05, 234.86/231.57 and 236.07/233.03 eV, respectively. In addition, one peak for S 2s is observed at 226.4 eV, which specifies the presence of bridging S^2−^.^36^ The two peaks at binding energies of 161.8 eV and 163 eV are detected for S 2p, which can be attributed to S 2p_3/2_ and 2p_1/2,_ respectively, as shown in Fig. 2(f). One minor peak for S at a higher binding energy of 168.52 eV appeared due to surface oxidation of MoS_2_ sheets.^37^

### FESEM and TEM of CdMoS_4_

The surface morphology of CdMoS_4_ samples is shown in Fig. 3(a–c). Fig. 3(a and b) shows the FESEM images that clearly show the formation of a porous nanoflower-like surface morphology of the CdMoS_4_ sample. The average size of the nanoflower was observed to be ∼3 µm and thickness ∼50 nm. This kind of porous nano flower-like structure of CdMoS_4_ provides a fair amount of surface area, thus enabling easy transportation of generated electron–hole to the surface and enhancing the photodetector activity. Fig. 3(c) shows the TEM image for the as-prepared CdMoS_4_ sample. An original CdMoS_4_ nanoflower in Fig. 3(c) shows a marigold flower-like microstructure of ∼2 µm. The uniform hierarchical nanostructures consist of well-organized independent nano petals with a length of 20–30 nm (Fig. 3(c)).

### XPS of CdMoS_4_

Fig. 3(d–f) present the XPS spectra for Cd 3d, Mo 3d and S 2p elements of CdMoS_4_ annealed at 400 °C. The Cd 3d doublet peaks at 413.4 eV and 406.6 eV are observed for Cd 3d_3/2_ and 3d_5/2_, respectively, as shown in Fig. 3(d). Fig. 3(e) displays two prominent peaks at 232.24 eV and 229.1 eV for Mo 3d, indicating the presence of a +4 oxidation state for Mo. The S 2p spectra in Fig. 3(f) also show two peaks at 163.1 eV and 161.8 eV, assigned to S 2p_1/2_ and S 2p_3/2_, respectively. Our XPS results match well with the earlier reports and further confirm the existence of Cd, Mo and S in CdMoS_4_ nanoflowers. It is apparent that the surface of CdMoS_4_ is made up of comparatively smooth sheets.

The UV-visible spectroscopy has been carried out for MoS_2_ and CdMoS_4_ annealed samples to investigate the optical properties presented (see in ESI Fig. S1†). The red plot shows the broad absorption for CdMoS_4_ nanoflowers in the UV region compared to MoS_2_ (black color).

### Photoresponse study of MoS_2_

**Fig. 4:**
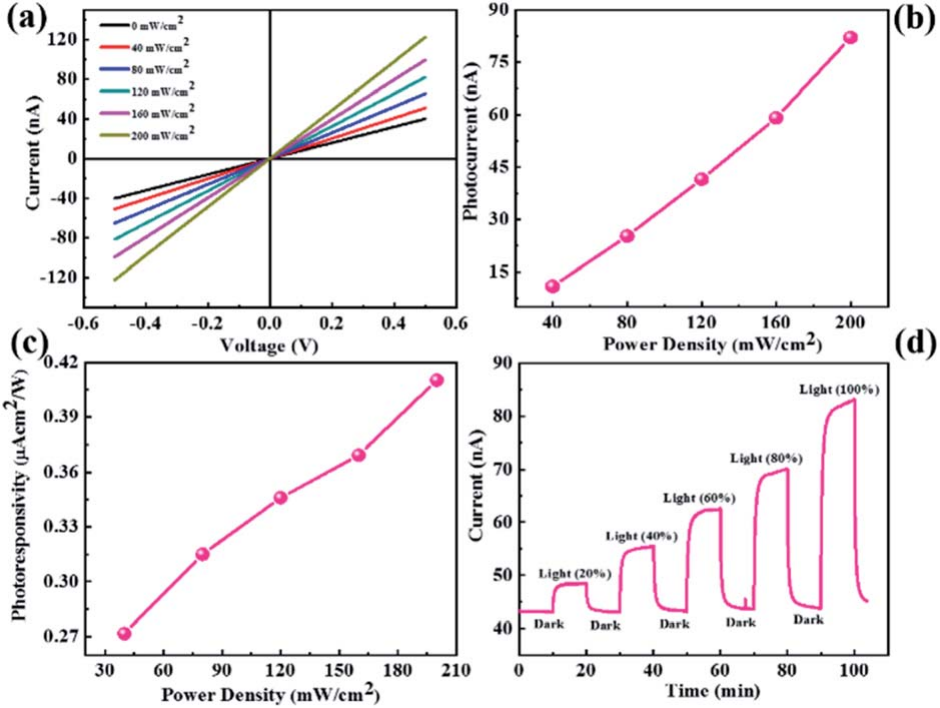
MoS_2_ nanosheet-based photodetector device. (a) current–voltage (*I*–*V*) characteristics, (b) photocurrent as a function of power density, (c) photoresponsivity *vs.* power density and (d) current–time (*I*–*t*) plot.

The as-synthesized MoS_2_ and CdMoS_4_ samples were further used for UV light photodetection. The experimental setup used for the measurement of UV light photodetection is shown in ESI Fig. S2.†Fig. 4(a) shows the *I*–*V* characteristics of the MoS_2_ nanosheet sensor device for numerous power densities under UV light ranging from 0–200 mW cm^−2^. We observed that the current increases with the increase in the power density of UV light. Photocurrents as a function of the power density plot are shown in Fig. 4(b), which indicates that the photocurrent increases with an increase in the power density. The photocurrent is calculated using the formula shown in eqn (1).

1 *I*_p_ = *I*_illumination_ − *I*_dark_

Fig. 4(c) shows the photoresponsivity *vs.* power density plot, whereby photoresponsivity is defined as the ratio of photocurrent to power density. We observed that the photoresponsivity increases with the increase in the power density. The photocurrent response of the MoS_2_ nanosheet photodetector is shown in Fig. 4(d), which is measured under light illumination and dark conditions at an applied bias voltage of 0.5 V. The response time and recovery time with the MoS_2_ nanosheet-based sensor were ∼118 s and ∼123 s, respectively.

### Photoresponse study of CdMoS_4_

**Fig. 5:**
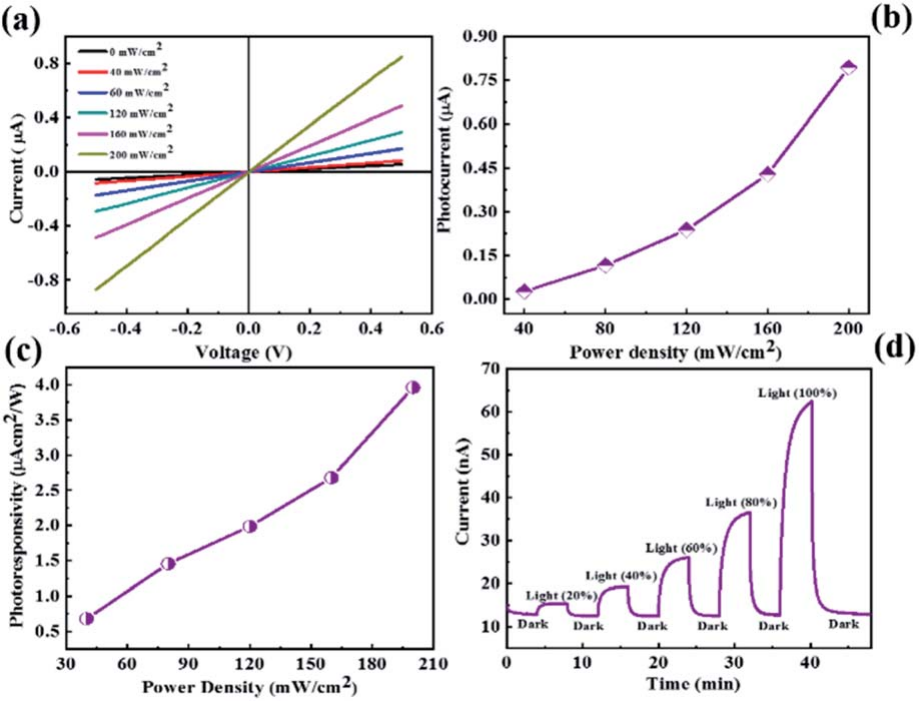
CdMoS_4 _nanoflower-based photodetector device (a) current–voltage (*I*–*V*) characteristics, (b) photocurrent as a function of power density, (c) photoresponsivity *vs.* power density and (d) current–time (*I*–*t*) plot.

Similarly, we have performed the UV light photodetector measurements for the CdMoS_4_ device shown in Fig. 5. Fig. 5(a) shows the *I*–*V* characteristics of the CdMoS_4_ nanosheet sensor device at various power densities of UV light ranging from 0–200 mW cm^−2^. We observed the increment in the current value with the increase in power density. The photocurrent as a function of power density is shown in Fig. 5(b). Upon increasing the power density, the photocurrent also increases. The photoresponsivity *vs.* power density plot is depicted in Fig. 5(c). The obtained maximum photoresponsivity was ∼4 µA cm^2^ W^−1^ at a power density of 200 mW cm^−2^, which is much higher than that of the MoS_2_-based device. The response time and recovery time for the CdMoS_4_-based device are calculated from the *I*–*t* plot shown in Fig. 5(d). The values are found to be ∼74 s and ∼94 s, respectively, which are lower than those of the device fabricated for the MoS_2_ nanosheet sample.

The proposed mechanism details under biasing and light illuminations using ITO electrodes, as shown in ESI Fig. S3(a) and (b).† The role of Cd was to produce impurity energy levels into the material, which shifts the absorption edges toward a longer wavelength, resulting in the enhancement in the properties of the photodetector. The enhanced photoexcited carrier density of the CdMoS_4_ sample suppressing the electron–hole recombination helps in improving the performance of the photodetector. In addition, the self-assembled nanoflowers from thin petals and the high surface area required for the absorption of light results in a higher number of charge carriers. The electron transfer becomes faster because of the thin petal-like morphology of the CdMoS_4_ sample, which also inhibits charge recombination. The comparative photodetector performance of MoS_2_ and CdMoS_4_ with previously reported Mo-based devices is presented in ESI Table 1.† The cyclic photoresponse study for the annealed MoS_2_ and CdMoS_4_ samples was carried out for more than 2000 s, and the various cycles have been recorded in light and dark conditions. The current–time (*I*–*t*) plots for the MoS_2_ and CdMoS_4_-based devices are shown in ESI Fig. S4(a) and (b),† respectively. Both the devices displays good photoresponse stability and reproducibility under UV light illumination. The results reveal that the CdMoS_4_ device showed good cyclic stability without showing any change in the photocurrent value compared to the MoS_2_ device for a long periods.

## Conclusion

In conclusion, the structural and morphological investigation of as-synthesized MoS_2_ nanosheets and CdMoS_4_ nanoflowers were investigated *via* spectroscopy and microscopy techniques. The UV photodetector devices were fabricated on ITO (indium tin oxide)-coated glass substrates. The CdMoS_4_ nanoflowers device showed a better response to UV light compared to pristine MoS_2_ nanosheets in terms of photoresponsivity, whereby the response time was 74 s and the recovery time was 94 s. Our results reveal that the device performance of CdMoS_4_ materials can be improved by making composite or heterostructures using other 2D materials or by functionalization.

## Author contributions

MSP has carried out the experimental work on device fabrication, analysis and first writing of the draft; SRK synthesized the materials and performed the XPS measurements; BBK and DJL supervised the overall work with comments on the first draft, writing, reviewing and editing.

## Conflicts of interest

There are no conflicts of interest to declare.

## Supplementary Material

NA-003-D1NA00326G-s001
