# Supplementary material for: MoS2 and CdMoS4 nanostructure-based UV light photodetectors
Source: Nanoscale Adv. 2021 Jul 21;3(16):4799–803. doi: 10.1039/d1na00326g (PMC9417253; doi:10.1039/d1na00326g)
Supplement: NA-003-D1NA00326G-s001 [file NA-003-D1NA00326G-s001.pdf]

Electronic Supplementary Material (ESI) for Nanoscale Advances.
This journal is © The Royal Society of Chemistry 2021

Supplementary Material

MoS_2 and $CdMoS_4$ nanostructures based UV light Photodetector

Mahendra S. Pawar,[a,b] Sunil R. Kadam,[c] Bharat B. Kale,[*c] Dattatray J. Late[*,a,b,d]

Fig. S1

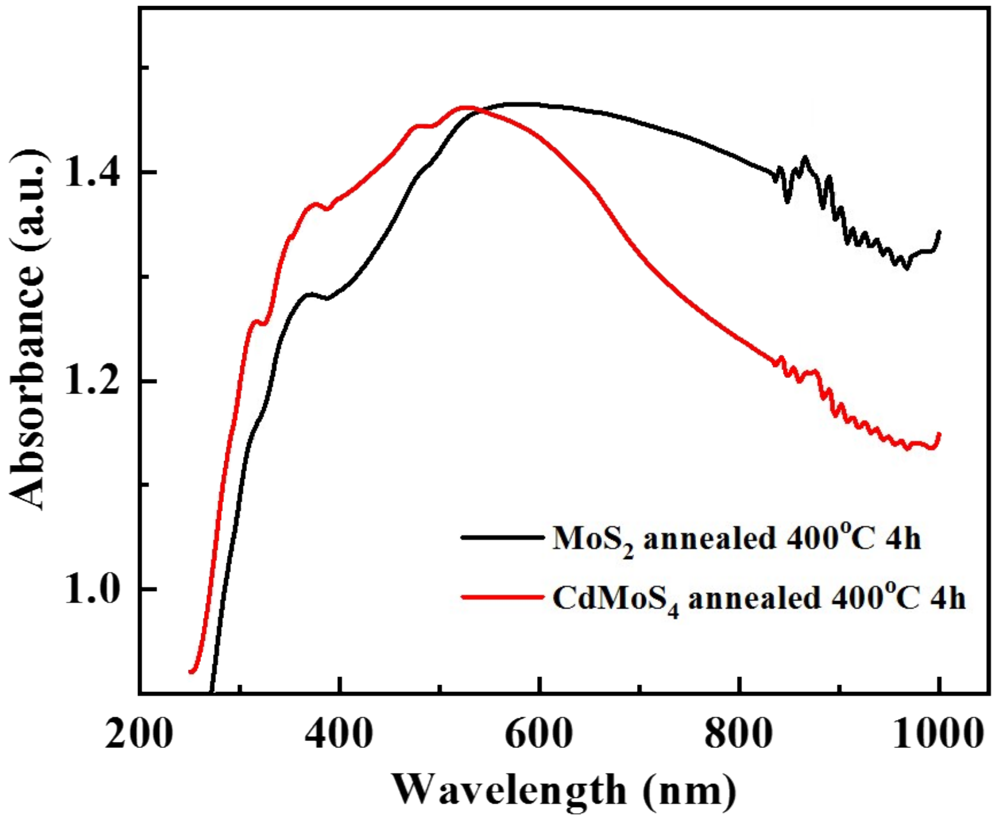

Fig. S1 UV-Visible spectra for MoS_2 (Black color) and $CdMoS_4$ (Red color) samples annealed at 400°C.

Fig. S2

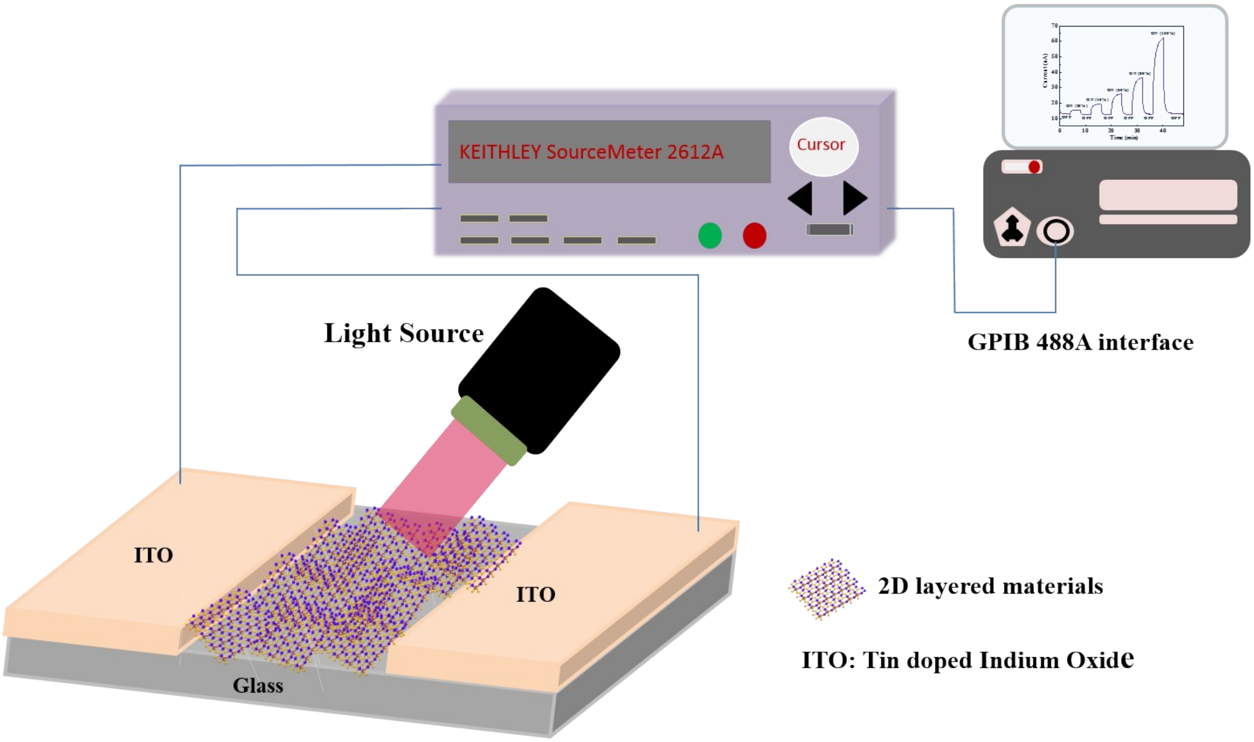

Fig. S2 Schematic of the Experimental setup for measurement of UV light Photodetector.

Fig. S3

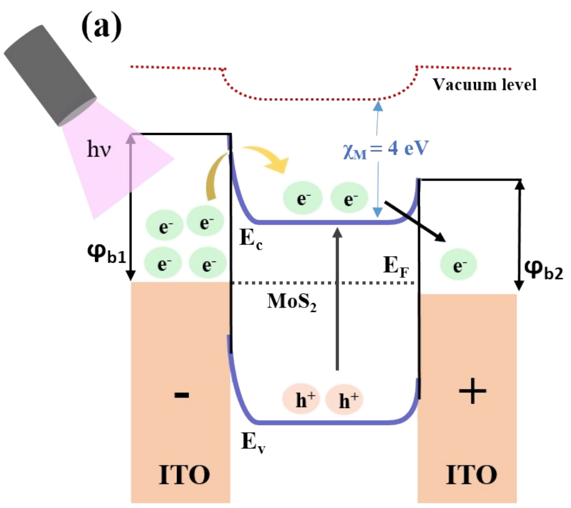

Fig. S3 Energy band diagram for (a) MoS_2 and (b) $CdMoS_4$using ITO electrode under biasing and light illumination conditions.

Photodetector mechanism under biasing and light illuminations using ITO electrode: The fig.S4 shows the energy band diagram for the Schottky barrier (ITO/MoS_2) and (ITO/$CdMoS_4$) in the photodetector device under biasing and light illumination conditions. The parameters such as φ_b (4.7 eV), χ_M (4 eV), φ_{b1} and φ_{b2} are the workfunction of the ITO electrode, electron affinity of the MoS_2, schottky barrier height at the ITO/MoS_2 and ITO/$CdMoS_4$ respectively.[1] When light falls on the device under biasing conditions electrons from the ITO layer will start crossing the schottky barrier (φ_{b1}). Once the barrier is crossed due to thermionic field emission it easily crosses the lower schottky barrier (φ_{b2}), further these electrons are collected at the positively biased Ito electrode. In case of $CdMoS_4$ the barrier height is lower compared to MoS_2 hence it results in the generation and collection of charge carriers. This gives rise to enhancement in the photocurrent value also the photoresponsivity.

Fig. S4

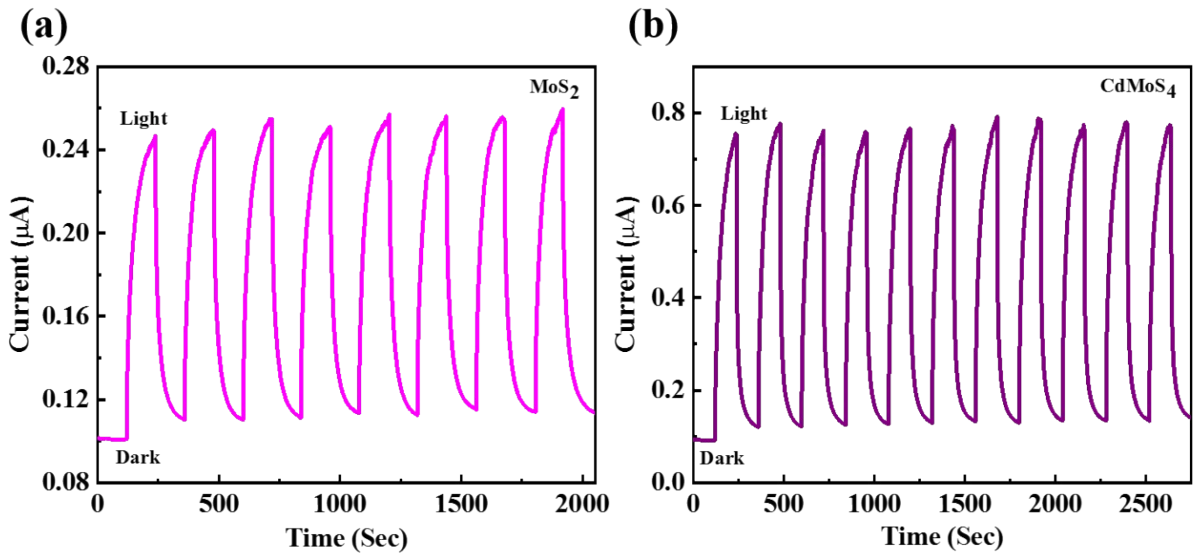

Fig. S4 Cyclic photo response study on (a) MoS_2 and (b) $CdMoS_4$ samples annealed at 400^0C.

Table. 1

Material	Spectral Range (nm)	Responsivity	Response Time	Recovery Time	Ref
MoS_2	1550	47.5 mA/W	10 ms	16 ms	2
MoS_2/Black Phosphorous	1550	153.4 mA/W	15 µs	70 µs	3
Bilayer MoS_2	1070	5.2 A/W	44.5 s	404.7 s	4
rGO/MoS_2		2.1 A/W	18 ms		5
MoS_2	637	~1 A/W	64 µs	51 µs	6
$MoS_{2.19}$	THz radiation	10 mA/W	5.12 s	6.33 s	7
Few layer MoS_2	532	~20 mA/W	12 s	19 s	8
MoS_2/$CsPbBr_3$	442	4.4 A/W	0.72 ms	1.01 ms	9
MoS_2/CdTe	780	36.6 mA/W	43.7 µs	82.1 µs	10
MoS_2	365	3 * 10^4 A/W	32 ms		11
MoS_2	385	0.41 µAcm^2/W	118 s	123 s	This work
$CdMoS_4$		4 µAcm^2/W	74 s	94 s	

Table 1: Comparative photodetector performance of the MoS_2 and $CdMoS_4$ to the previously reported Mo based photodetector devices.

References:

1) Y. Son, Q. H. Wang, J. A. Paulson,C. J. Shih,A. G. Rajan, K. Tvrdy, S. Kim, B. Alfeeli, R. D. Braatz, and M. S. Strano, *ACS Nano* 2015, **9**, 2843.
2) Z. Huang, T. Zhang, J. Liu, L. Zhang, Y. Jin, J. Wang, K. Jiang, S. Fan, and Q. Li, *ACS Appl. Electron. Mater*. 2019, **1**, 1314.
3) L. Ye, H. Li, Z. Chen, and J. Xu*, ACS Photonics* 2016, **3**, 692.
4) W. Wang, A. Klots, D. Prasai, Y. Yang, K. I. Bolotin, and J. Valentine, *Nano Lett.* 2015, **15**, 7440.
5) R. Kumar, N. Goel, R. Raliya, P. Biswas, and M. Kumar, *Nanotechnology* 2018, **29**, 404001.
6) F. Gong, H. Fang, P. Wang, M. Su, Q. Li, J. C. Ho, X. Chen, W. Lu, L. Liao, J. Wang, and W. Hu, 2017. *Nanotechnology* 2017, **28**, 484002.
7) Y. Xie, F. Liang, S. Chi, D. Wang, K. Zhong, H. Yu, H. Zhang, Y. Chen, and J. Wang, 2020. *ACS Appl. Mater. Interfaces* 2020, **12**, 7351.

8) F. Yu, M. Hu, F. Kang, and R. Lv, *Prog. Nat. Sci.: Mater. Int*. 2018, **28**, 563-568.
9) X. Song, X. Liu, D. Yu, C. Huo, J. Ji, X. Li, S. Zhang, Y. Zou, G. Zhu, Y. Wang, and M. Wu, *ACS Appl. Mater. Interfaces* 2018, **10**, 2801.
10) Y. Wang, X. Huang, D. Wu, R. Zhuo, E. Wu, C. Jia, Z. Shi, T. Xu, Y. Tian, and X. Li, *J. Mater. Chem. C* 2018, **6**, 4861.
11) S. Kumar, A. Sharma, Y. T. Ho, A. Pandey, M. Tomar, A. K. Kapoor, E. Y. Chang, and V. Gupta, *J. Alloys Compd*. 2020, **835**, 155222.
